# Supplementary material for: Experimental Evolution of Yeast Reveals Trade-offs Between Early and Late Stationary Phase
Source: bioRxiv. 2026 Mar 16:2026.03.12.711341. Preprint. [Version 2] doi: 10.64898/2026.03.12.711341 (PMC13015349; doi:10.64898/2026.03.12.711341)
Supplement: Supplement 1 [file NIHPP2026.03.12.711341v2-supplement-1.pdf]

bioRxiv preprint doi: https://doi.org/10.64898/2026.03.12.711341; this version posted March 16, 2026. The copyright holder for this preprint (which was not certified by peer review) is the author/funder, who has granted bioRxiv a license to display the preprint in perpetuity. It is made available under aCC-BY 4.0 International license.

Supplemental Figures

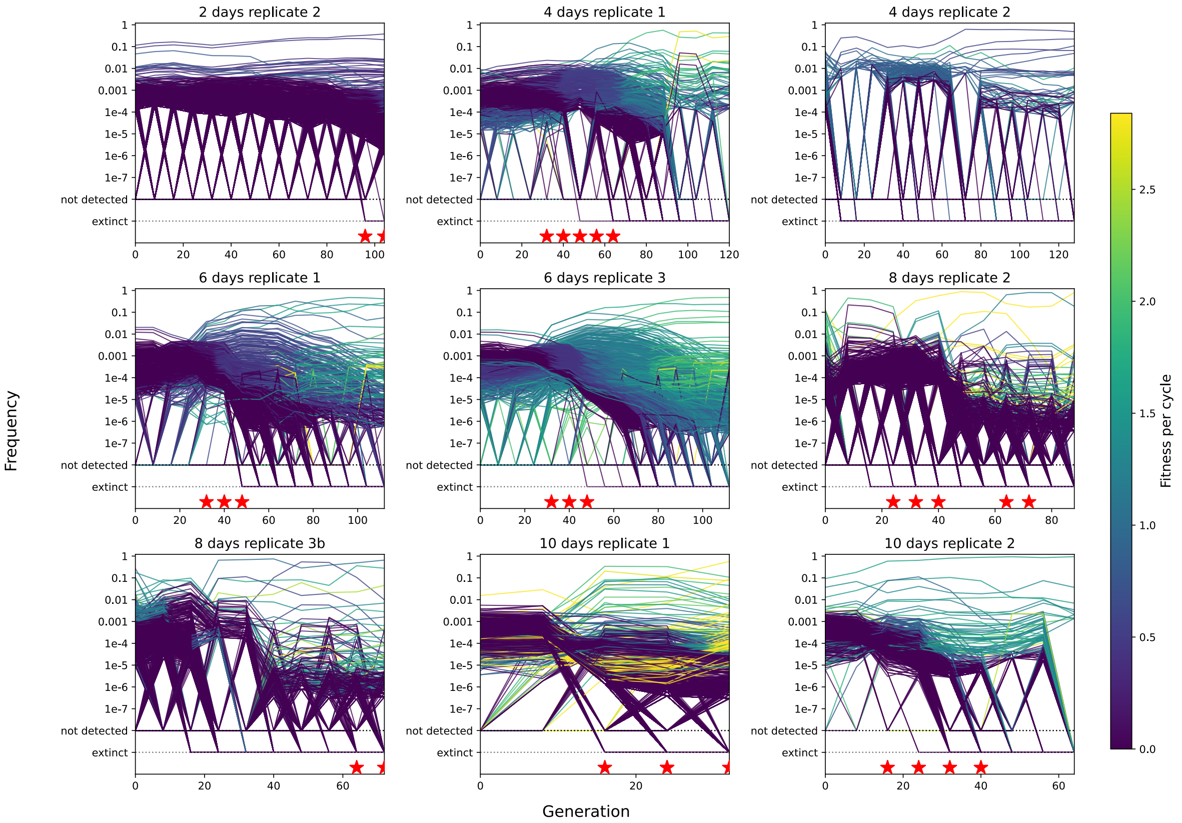

Figure S1. Evolutionary trajectories of a subset of Gly/Eth evolved populations. Each line shows the trajectory of a barcoded lineage colored based on the fitness inferred by FitMut2. Lineages are plotted as extinct when they are not detected in a timepoint and are also undetectable in all subsequent timepoints. Red stars indicate the timepoints from which clones were sampled.

bioRxiv preprint doi: https://doi.org/10.64898/2026.03.12.711341; this version posted March 16, 2026. The copyright holder for this preprint (which was not certified by peer review) is the author/funder, who has granted bioRxiv a license to display the preprint in perpetuity. It is made available under aCC-BY 4.0 International license.

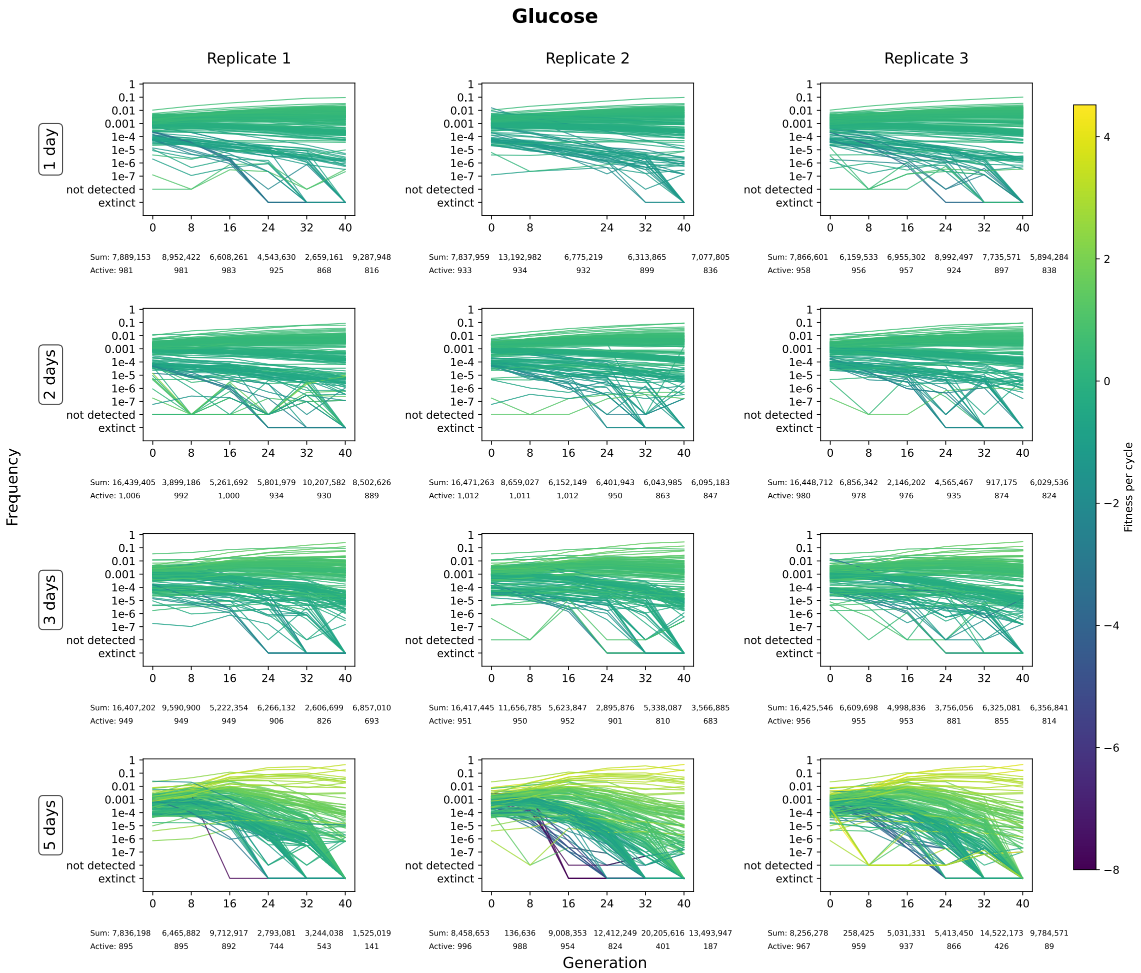

bioRxiv preprint doi: https://doi.org/10.64898/2026.03.12.711341; this version posted March 16, 2026. The copyright holder for this preprint (which was not certified by peer review) is the author/funder, who has granted bioRxiv a license to display the preprint in perpetuity. It is made available under aCC-BY 4.0 International license.

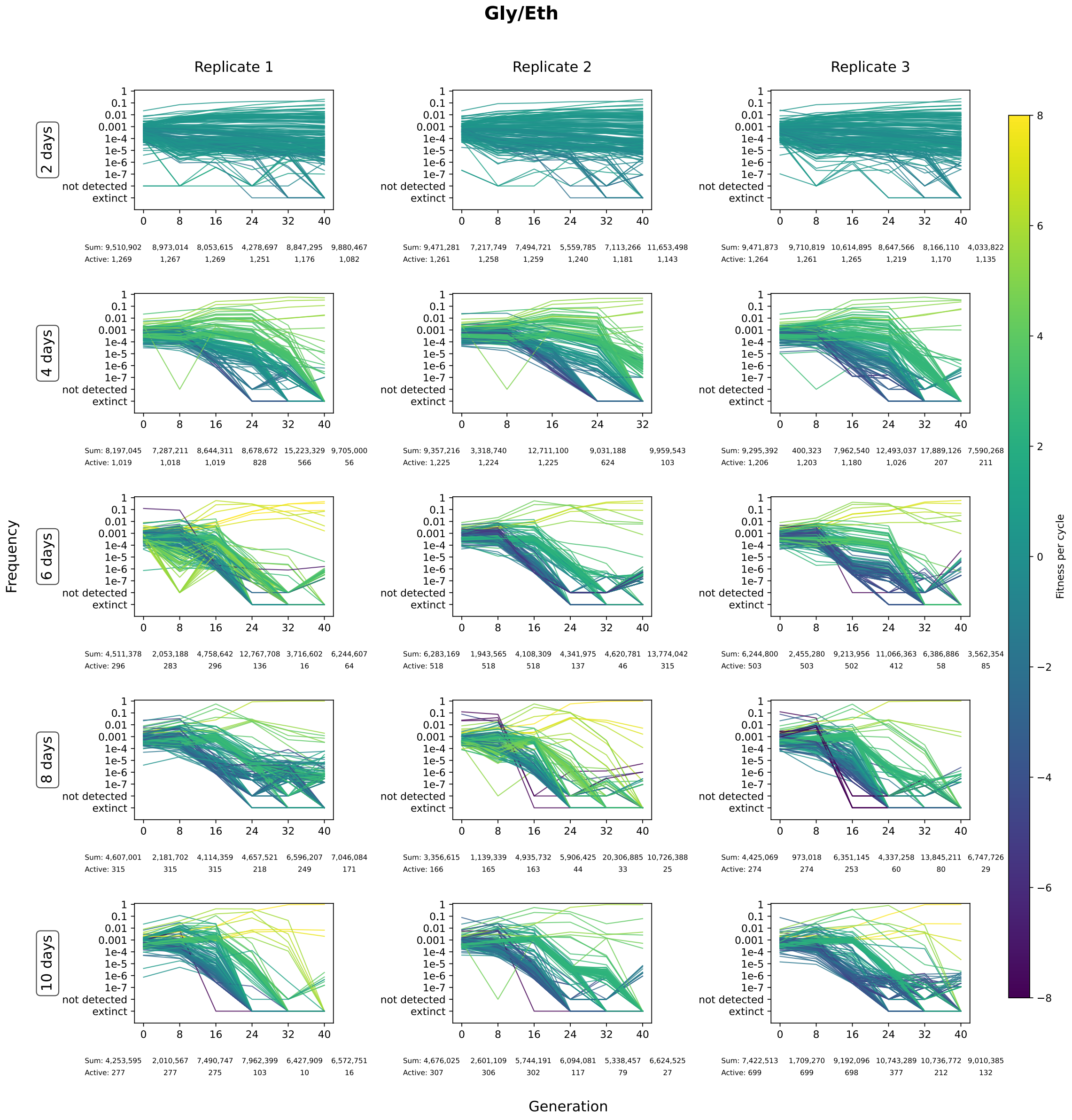

Figure S2. Fitness remeasurement trajectories. Trajectories of a subset of barcodes in the remeasurement pool are shown for all 3 replicates measured in 9 conditions. The top 100 fittest lineages and 100 randomly selected lineages are plotted for each replicate. Only lineages for which fitness is estimated with an error of <5 are plotted. Lineages are colored by the fitness inferred by FitSeq2. Sum indicates the total number of counts in each timepoint and Active indicates the number of non-zero unique barcodes in each timepoint.

bioRxiv preprint doi: https://doi.org/10.64898/2026.03.12.711341; this version posted March 16, 2026. The copyright holder for this preprint (which was not certified by peer review) is the author/funder, who has granted bioRxiv a license to display the preprint in perpetuity. It is made available under aCC-BY 4.0 International license.

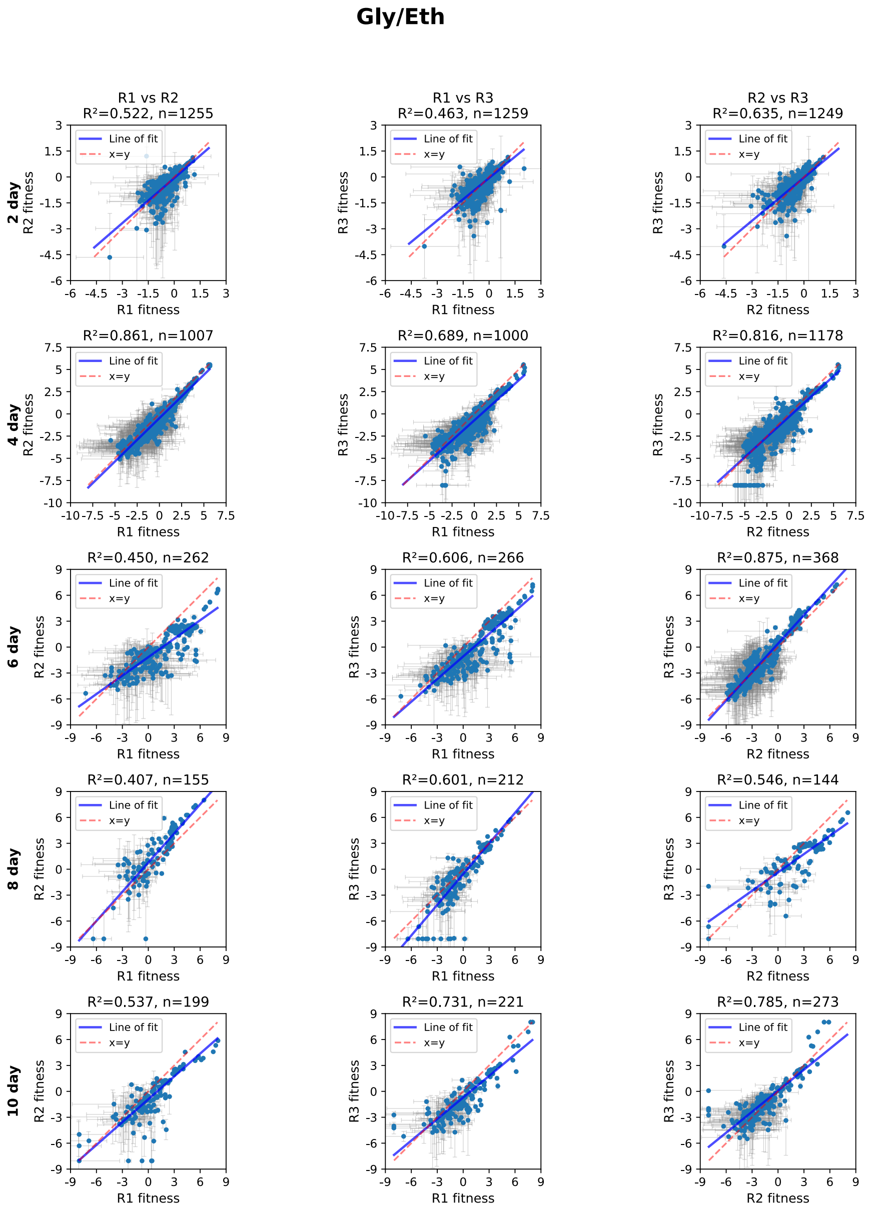

bioRxiv preprint doi: https://doi.org/10.64898/2026.03.12.711341; this version posted March 16, 2026. The copyright holder for this preprint (which was not certified by peer review) is the author/funder, who has granted bioRxiv a license to display the preprint in perpetuity. It is made available under aCC-BY 4.0 International license.

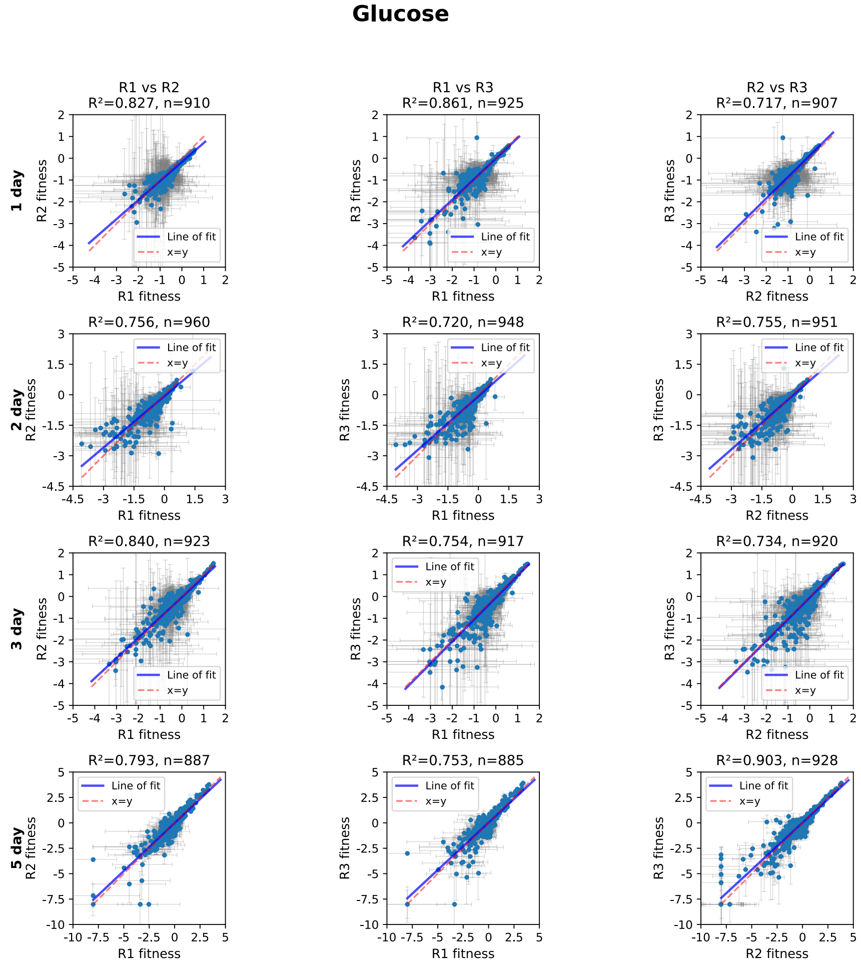

Figure S3. Replicate-replicate fitness correlations. For each assay condition replicate-replicate correlations are shown for fitness per cycle inferred by FitSeq2. Error bars shown in grey are estimated by FitSeq2. Only measurements with error less than 5 units are included in the plots and downstream analysis.

bioRxiv preprint doi: https://doi.org/10.64898/2026.03.12.711341; this version posted March 16, 2026. The copyright holder for this preprint (which was not certified by peer review) is the author/funder, who has granted bioRxiv a license to display the preprint in perpetuity. It is made available under aCC-BY 4.0 International license.

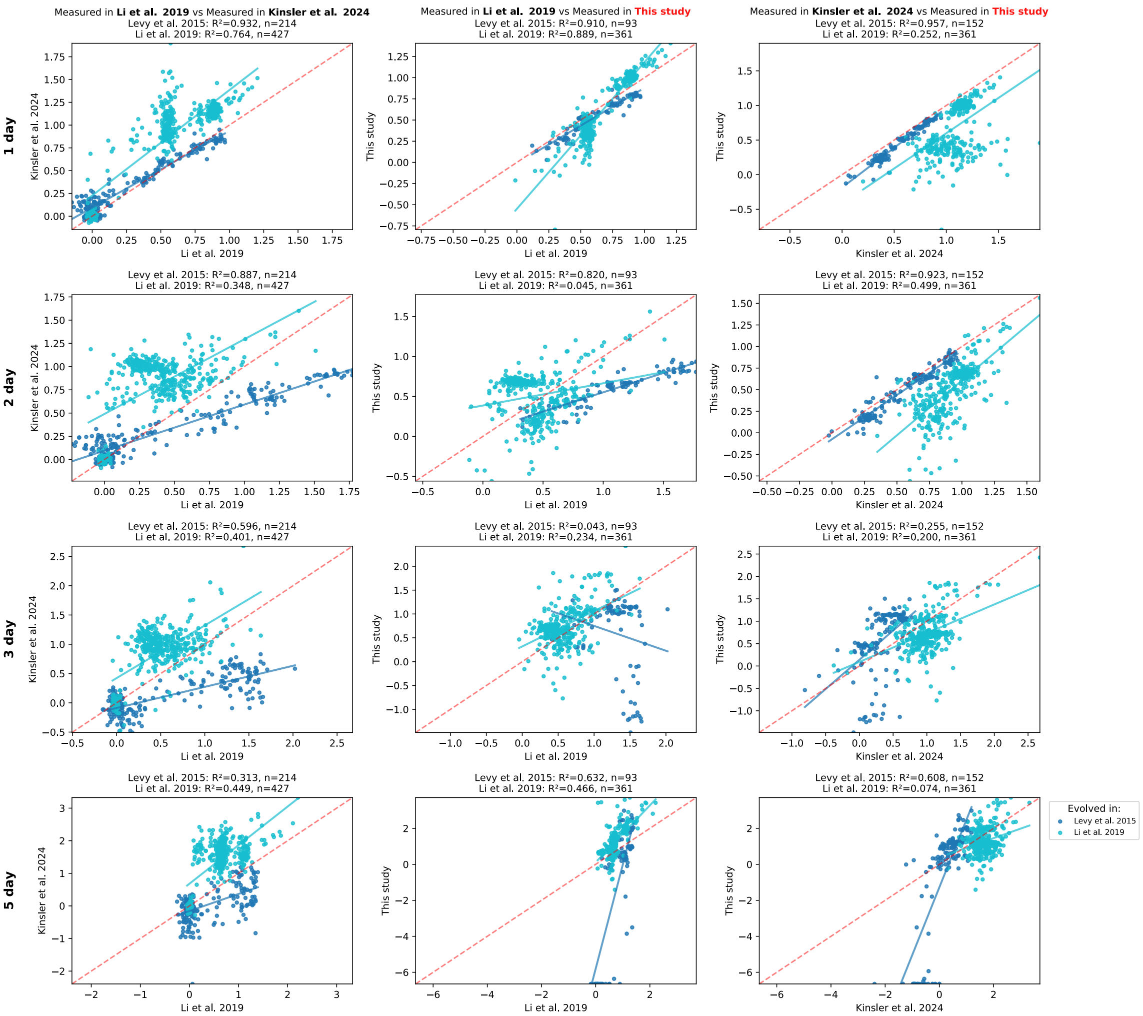

Figure S4. Measurements of glucose evolved clones in this study are comparable with previous measurements. Many of the glucose evolved clones included in our fitness remeasurement pool have been assayed previously in glucose conditions in Li et al. 2019 and Kinsler et al. 2024. The blue points show 2 day evolved glucose clones (Levy et al. 2015) and the cyan points show 1, 5, and 1/5 day glucose evolved clones (Li et al. 2019).

bioRxiv preprint doi: https://doi.org/10.64898/2026.03.12.711341; this version posted March 16, 2026. The copyright holder for this preprint (which was not certified by peer review) is the author/funder, who has granted bioRxiv a license to display the preprint in perpetuity. It is made available under aCC-BY 4.0 International license.

Gene	Gly/Eth					5 day Gluc.
	2 day	4 day	6 day	8 day	10 day	
IRA1	15	1	2	0	0	0
IRA2	17	1	3	0	2	1
PDE1	0	0	0	0	0	0
PDE2	8	0	0	0	0	0
GPB1	1	0	0	0	0	0
GPB2	8	0	0	1	2	0
YAK1	7	3	1	0	1	0
RAS1	0	0	0	0	0	1
RAS2	0	0	0	0	1	0
CYR1	1	0	0	0	0	1
TPK1	1	0	0	0	0	0
MSN4	1	0	0	0	0	0
BCY1	1	0	0	0	0	0
GPA2	0	0	0	0	1	0
Clones with sufficient coverage in WGS (see Methods)	**86**	**81**	**179**	**49**	**54**	**71**

Table S1. Ras/PKA mutations are less common in evolutions that include a stationary phase. The number of mutations in genes in the Ras/PKA pathway in clones isolated from different evolutionary conditions is shown.

bioRxiv preprint doi: https://doi.org/10.64898/2026.03.12.711341; this version posted March 16, 2026. The copyright holder for this preprint (which was not certified by peer review) is the author/funder, who has granted bioRxiv a license to display the preprint in perpetuity. It is made available under aCC-BY 4.0 International license.

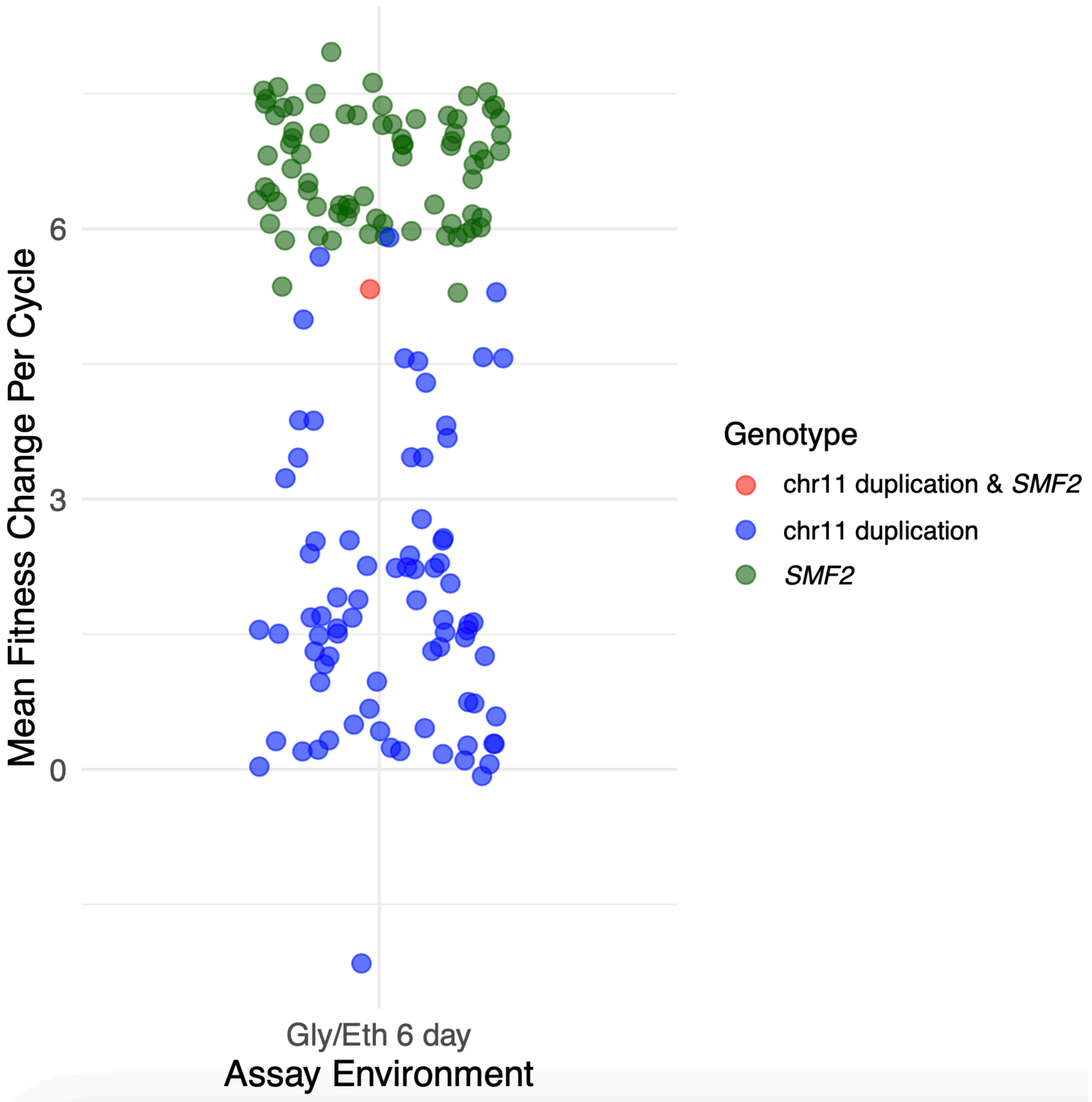

Figure S5. The *SMF2* mutants with a chromosome 11 duplication have lower fitness than other *SMF2* mutants. The Gly/Eth 6 day fitness of all evolved clones with a chromosome 11 duplication and/or an *SMF2* mutation. The chromosome 11 duplication does not provide any additional benefit in the *SMF2* mutant background.

bioRxiv preprint doi: https://doi.org/10.64898/2026.03.12.711341; this version posted March 16, 2026. The copyright holder for this preprint (which was not certified by peer review) is the author/funder, who has granted bioRxiv a license to display the preprint in perpetuity. It is made available under aCC-BY 4.0 International license.

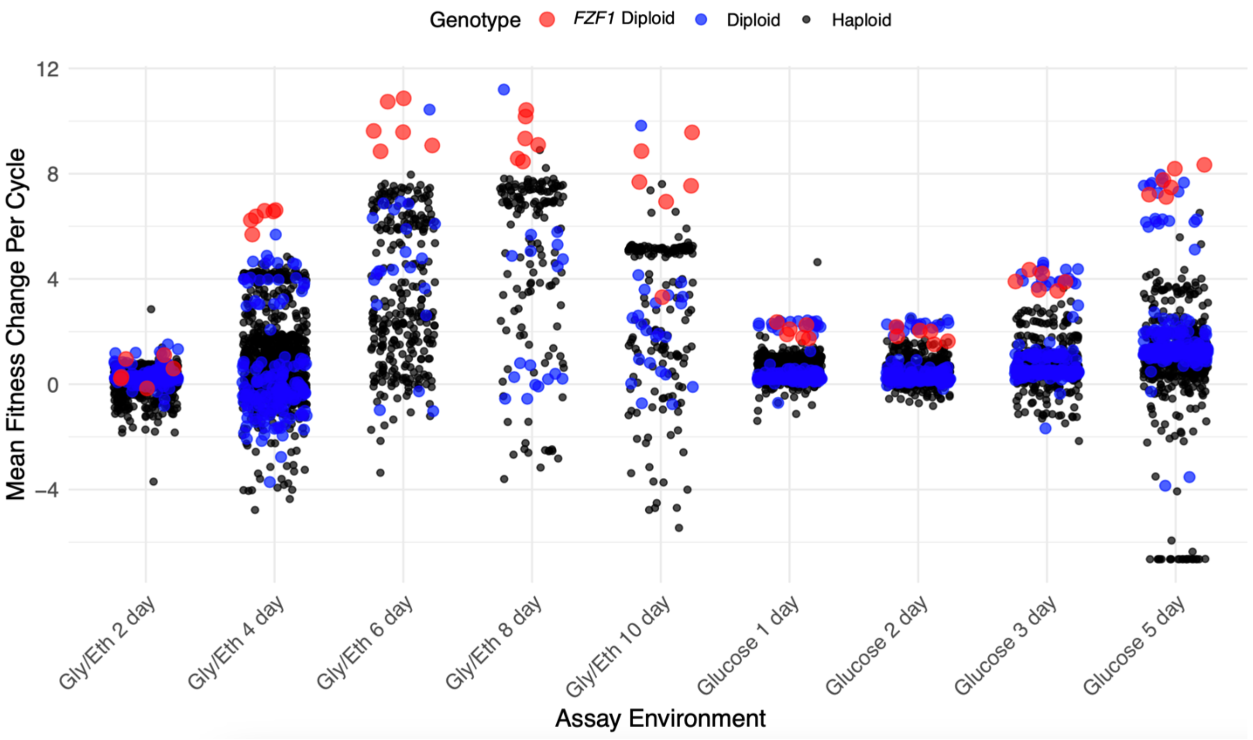

Figure S6. *FZF1* mutations are among the fittest in all conditions with a stationary phase. The mean fitness change per cycle of all adaptive mutants is plotted for each of the remeasurement conditions. The *FZF1* mutants, all of which are diploid, are colored red and perform particularly well in Gly/Eth conditions with stationary phase. Note that one of the *FZF1* clones was poorly measured and is not included in the plot.

bioRxiv preprint doi: https://doi.org/10.64898/2026.03.12.711341; this version posted March 16, 2026. The copyright holder for this preprint (which was not certified by peer review) is the author/funder, who has granted bioRxiv a license to display the preprint in perpetuity. It is made available under aCC-BY 4.0 International license.

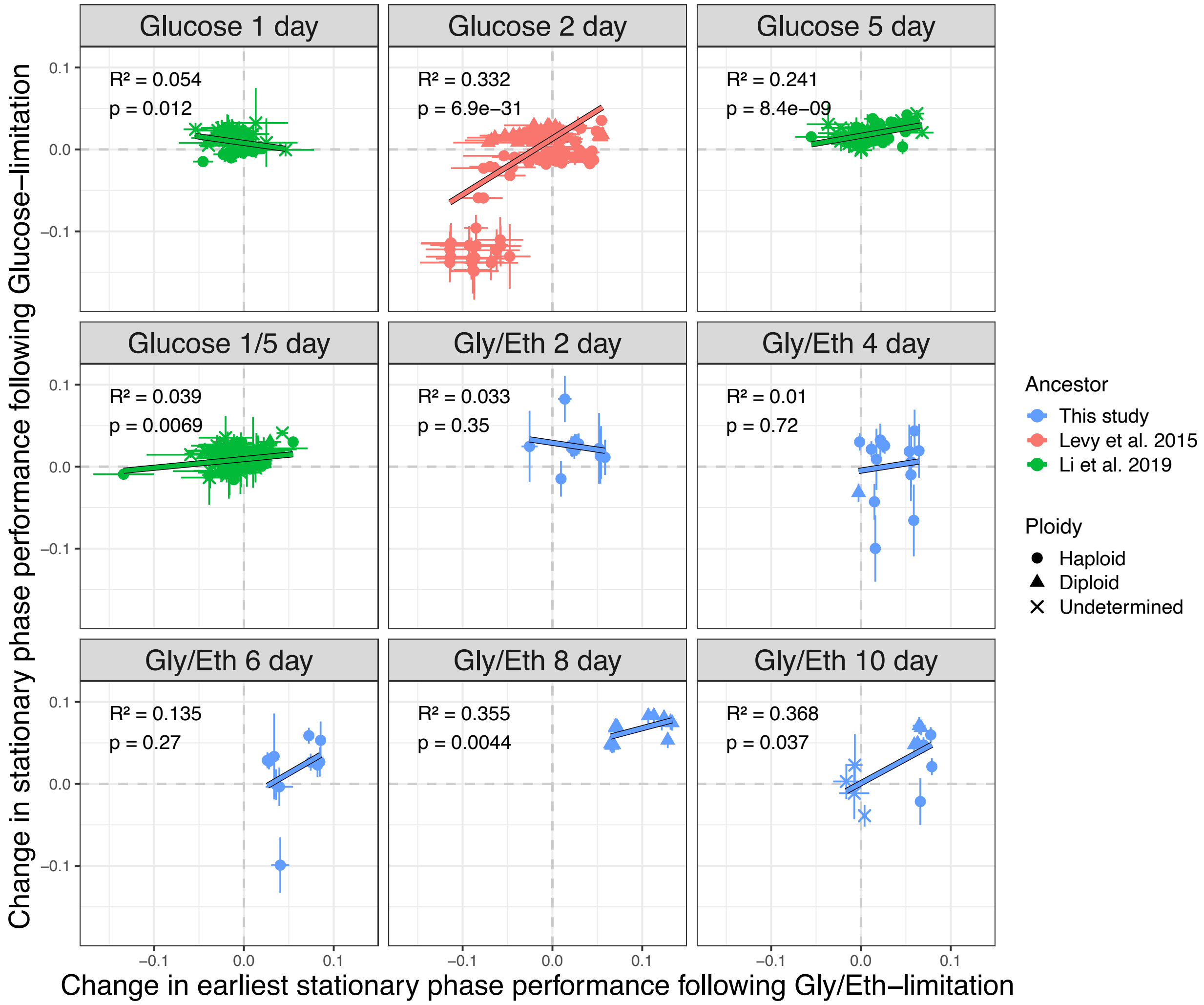

Figure S7. Stationary phase performance is correlated following glucose and Gly/Eth-limitation for clones that evolved with a stationary phase. Change in stationary phase performance (day 3-5) following glucose-limitation are correlated with changes in stationary phase performance (day 2-4) following Gly/Eth-limitation. For each evolution condition that included stationary phase, we observed a positive correlation between stationary phase performance following glucose and Gly/Eth-limitation.

bioRxiv preprint doi: https://doi.org/10.64898/2026.03.12.711341; this version posted March 16, 2026. The copyright holder for this preprint (which was not certified by peer review) is the author/funder, who has granted bioRxiv a license to display the preprint in perpetuity. It is made available under aCC-BY 4.0 International license.

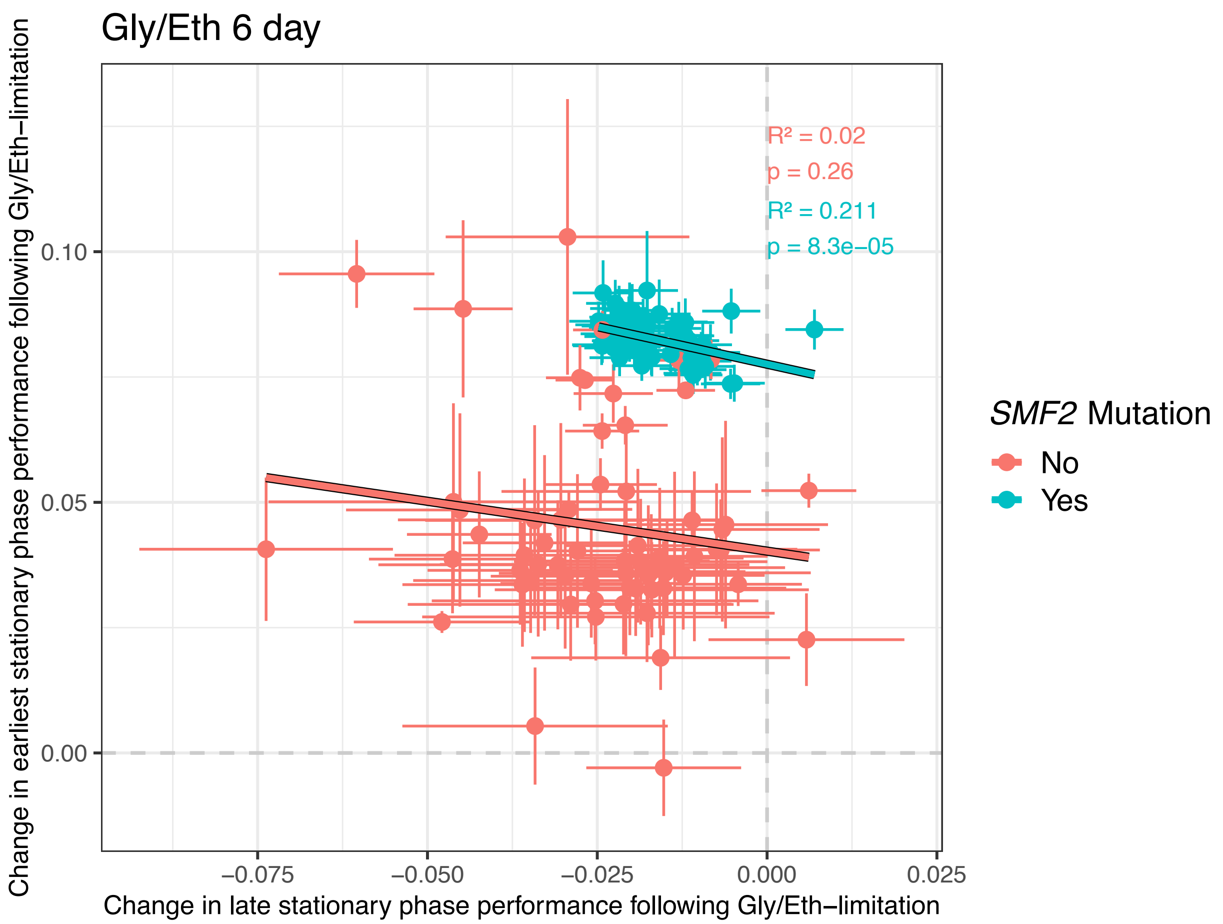

Figure S8. *SMF2* mutants cluster in trait-space compared to other adaptive mutants. The change in earliest (day 2-4) and late (day 6-10) stationary phase following Gly/Eth-limitation is shown for Gly/Eth 6-day adaptive haploid mutants. Clones in which an *SMF2* mutation was detected are colored turquoise and all other adaptive clones are shown in red. Results of a Pearson correlation test are shown for each group of adaptive mutants. Error bars indicate the combined error inferred by Fitseq2 for each underlying fitness measurement.

bioRxiv preprint doi: https://doi.org/10.64898/2026.03.12.711341; this version posted March 16, 2026. The copyright holder for this preprint (which was not certified by peer review) is the author/funder, who has granted bioRxiv a license to display the preprint in perpetuity. It is made available under aCC-BY 4.0 International license.

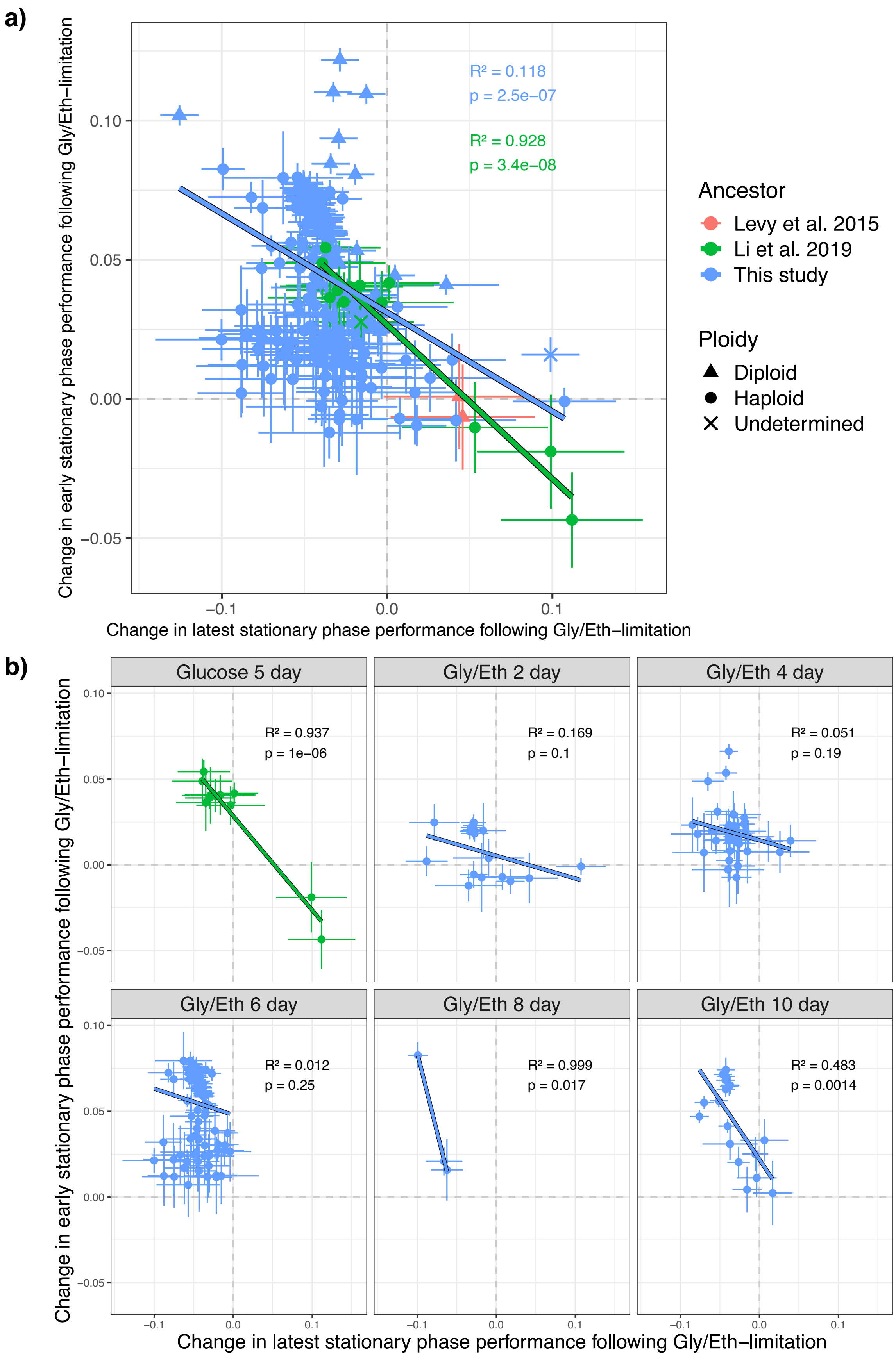

bioRxiv preprint doi: https://doi.org/10.64898/2026.03.12.711341; this version posted March 16, 2026. The copyright holder for this preprint (which was not certified by peer review) is the author/funder, who has granted bioRxiv a license to display the preprint in perpetuity. It is made available under aCC-BY 4.0 International license.

Figure S9. A trade-off between early and latest stationary phase performance. Adaptive haploids are plotted. A linear regression is shown through each set of all adaptive mutants comparing changes in early stationary phase performance (day 2-6) to latest stationary phase performance (day 8-10). (b) A linear regression is shown for adaptive haploid clones isolated from each evolution condition. Points are colored by the ancestor. Results of a Pearson correlation test are shown for each evolved group of adaptive haploid mutants. Error bars indicate the combined error inferred by Fitseq2 for each underlying fitness measurement.

bioRxiv preprint doi: https://doi.org/10.64898/2026.03.12.711341; this version posted March 16, 2026. The copyright holder for this preprint (which was not certified by peer review) is the author/funder, who has granted bioRxiv a license to display the preprint in perpetuity. It is made available under aCC-BY 4.0 International license.

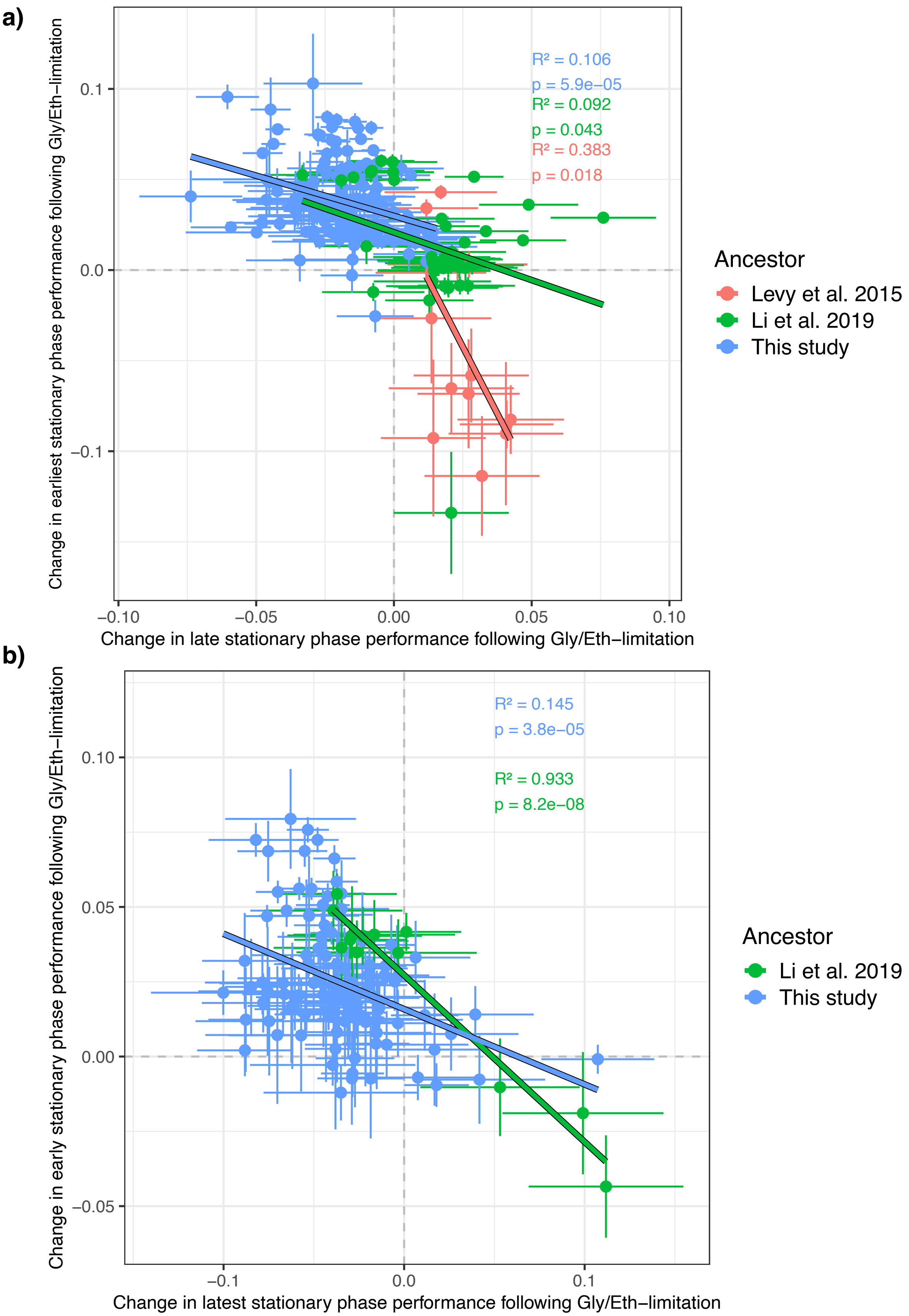

bioRxiv preprint doi: https://doi.org/10.64898/2026.03.12.711341; this version posted March 16, 2026. The copyright holder for this preprint (which was not certified by peer review) is the author/funder, who has granted bioRxiv a license to display the preprint in perpetuity. It is made available under aCC-BY 4.0 International license.

Figure S10. A trade-off between late and early stationary phase performance for adaptive haploids without *SMF2* mutations. (a-b) A linear regression is shown through each set of adaptive haploid mutants that lack the *SMF2* mutation comparing changes in early stationary phase performance (day 2-6) to latest stationary phase performance (day 8-10) (a) and comparing earliest stationary phase performance (day 2-4) to late stationary phase performance (day 6-10) (b). Results of a Pearson correlation test are shown for each group of adaptive mutants. Error bars indicate the combined error inferred by Fitseq2 for each underlying fitness measurement.

bioRxiv preprint doi: https://doi.org/10.64898/2026.03.12.711341; this version posted March 16, 2026. The copyright holder for this preprint (which was not certified by peer review) is the author/funder, who has granted bioRxiv a license to display the preprint in perpetuity. It is made available under aCC-BY 4.0 International license.

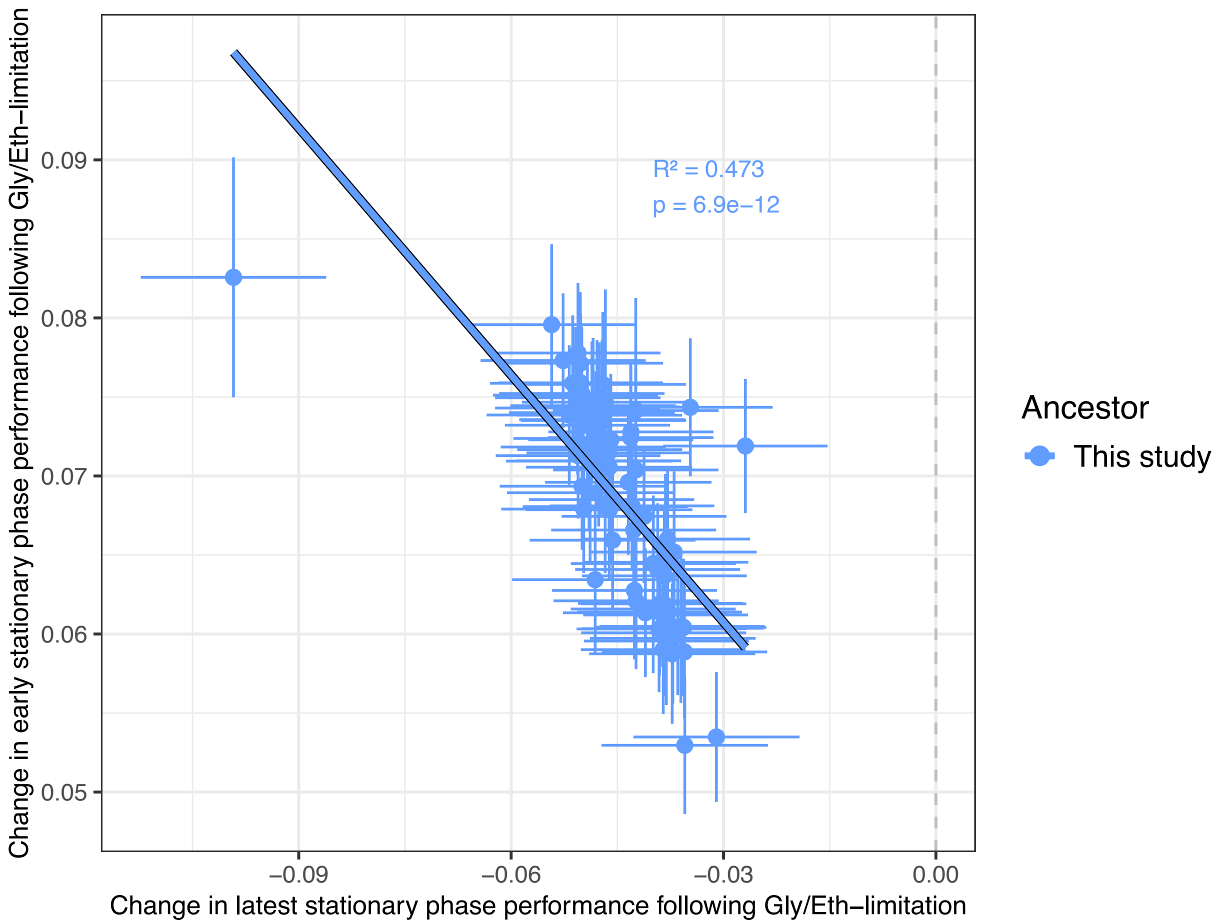

Figure S11. Trade-offs between early and late stationary phase performance among *SMF2* mutants. Changes in early stationary phase performance (day 2-6) following Gly/Eth-limitation are negatively correlated with changes in latest stationary phase (day 8-10) following Gly/Eth limitation. Only adaptive haploid mutants with a normal karyotype and an *SMF2* mutation are plotted. Results of a Pearson correlation test are shown.

bioRxiv preprint doi: https://doi.org/10.64898/2026.03.12.711341; this version posted March 16, 2026. The copyright holder for this preprint (which was not certified by peer review) is the author/funder, who has granted bioRxiv a license to display the preprint in perpetuity. It is made available under aCC-BY 4.0 International license.

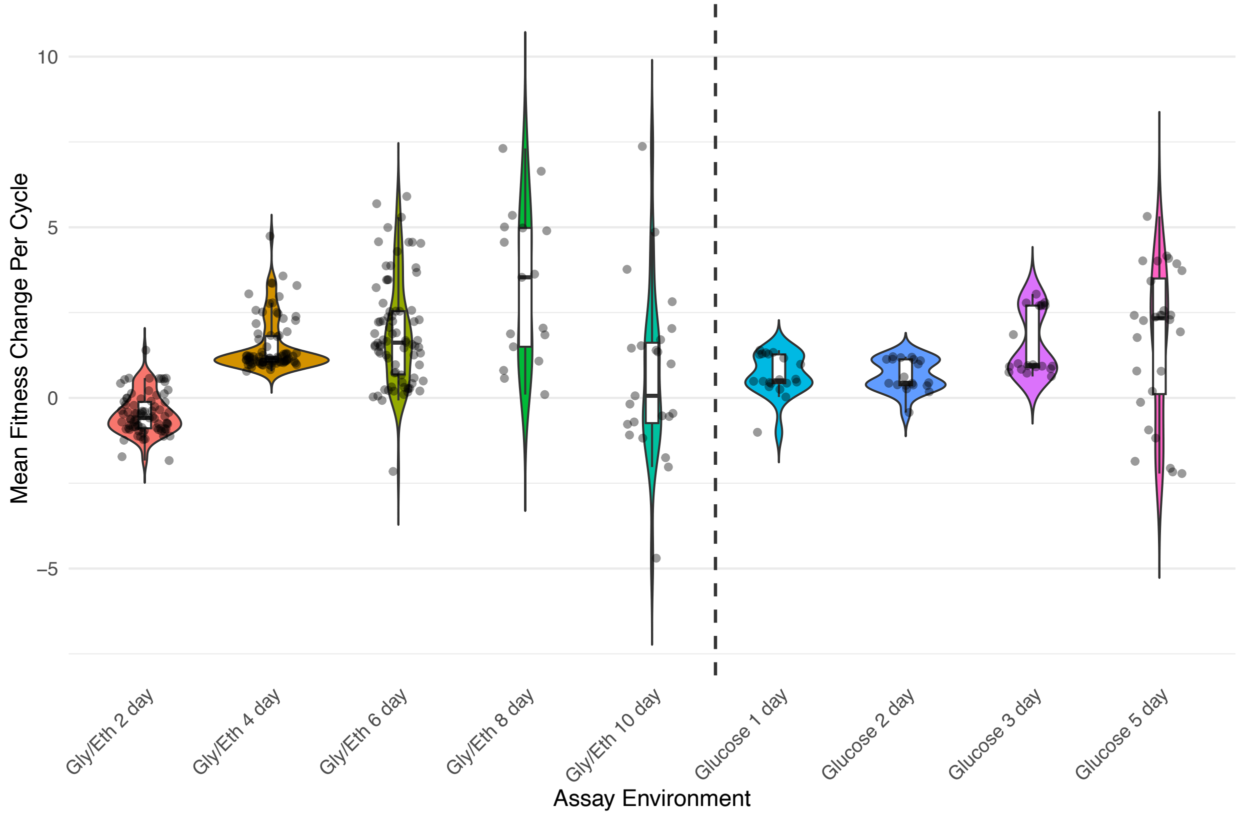

Figure S12. Most adaptive mutants with chromosome 11 duplications have increased fitness in the Gly/Eth 6 day condition. The change in mean fitness per cycle is plotted for all clones with a chromosome 11 duplication. These duplications are often deleterious in the 2-day and 10-day condition. There are fewer points plotted in some conditions (e.g. Gly/Eth 8 day and 10 day) because the clones drop in frequency in the fitness remeasurement assays resulting in the FitSeq2 fitness estimates having large error and not passing our filter.

bioRxiv preprint doi: https://doi.org/10.64898/2026.03.12.711341; this version posted March 16, 2026. The copyright holder for this preprint (which was not certified by peer review) is the author/funder, who has granted bioRxiv a license to display the preprint in perpetuity. It is made available under aCC-BY 4.0 International license.

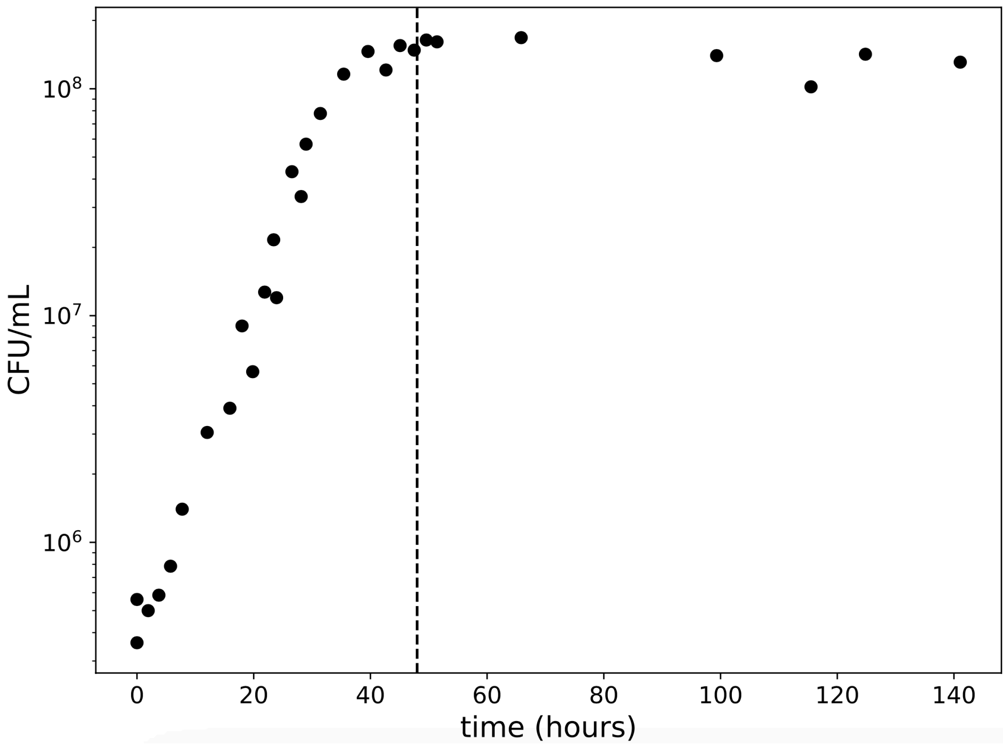

Figure S13. Barcoded yeast library reaches stationary phase after 48 hours. The barcoded yeast library was inoculated into 100 mL of Gly/Eth media and CFUs were measured by plating every ~2 hours. The dashed line shows the 48-hour timepoint after which the number of viable cells stops increasing.

bioRxiv preprint doi: https://doi.org/10.64898/2026.03.12.711341; this version posted March 16, 2026. The copyright holder for this preprint (which was not certified by peer review) is the author/funder, who has granted bioRxiv a license to display the preprint in perpetuity. It is made available under aCC-BY 4.0 International license.

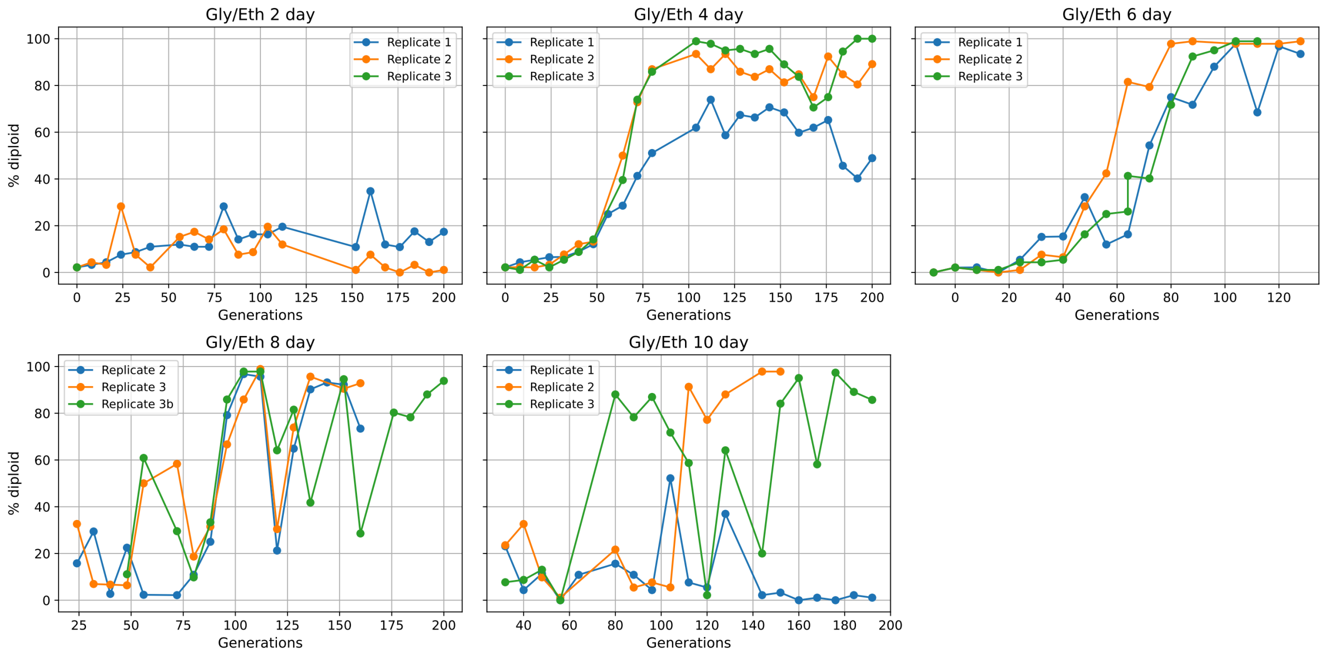

Figure S14. Diploids are selected during evolution in Gly/Eth. We measured the percent of cells that were diploid using benomyl assays as populations evolved. Each panel shows replicate populations from different evolution conditions.

bioRxiv preprint doi: https://doi.org/10.64898/2026.03.12.711341; this version posted March 16, 2026. The copyright holder for this preprint (which was not certified by peer review) is the author/funder, who has granted bioRxiv a license to display the preprint in perpetuity. It is made available under aCC-BY 4.0 International license.

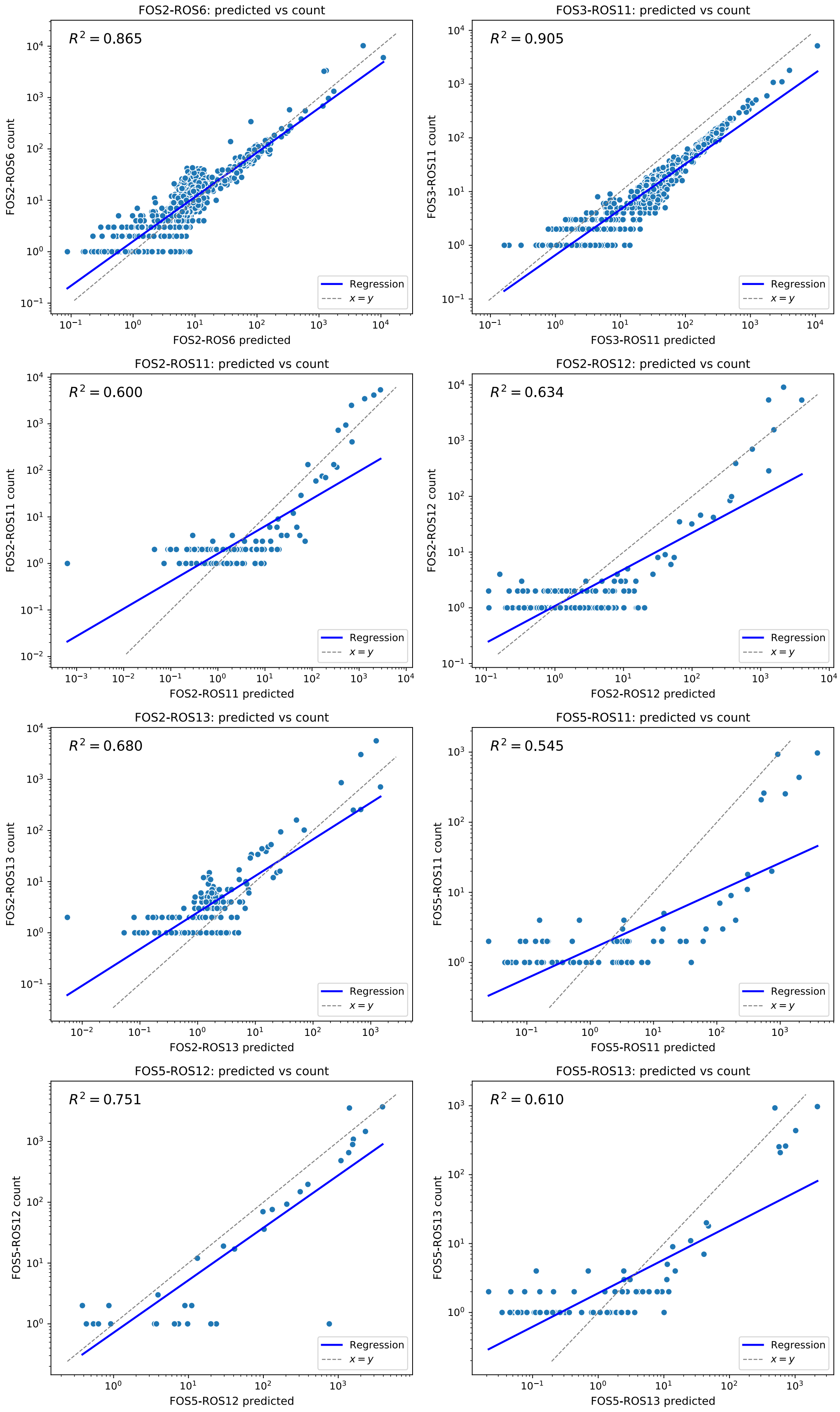

bioRxiv preprint doi: https://doi.org/10.64898/2026.03.12.711341; this version posted March 16, 2026. The copyright holder for this preprint (which was not certified by peer review) is the author/funder, who has granted bioRxiv a license to display the preprint in perpetuity. It is made available under aCC-BY 4.0 International license.

Figure S15. Generation of index hopped reads can be predicted. Eight FOS-ROS combinations were not included in the library preparation. These barcode counts are shown on the y-axis of each plot. The predicted number of BC for that FOS-ROS combination is shown on the x-axis. We corrected all counts to account for the generation of index hopped reads (see Methods).

bioRxiv preprint doi: https://doi.org/10.64898/2026.03.12.711341; this version posted March 16, 2026. The copyright holder for this preprint (which was not certified by peer review) is the author/funder, who has granted bioRxiv a license to display the preprint in perpetuity. It is made available under aCC-BY 4.0 International license.

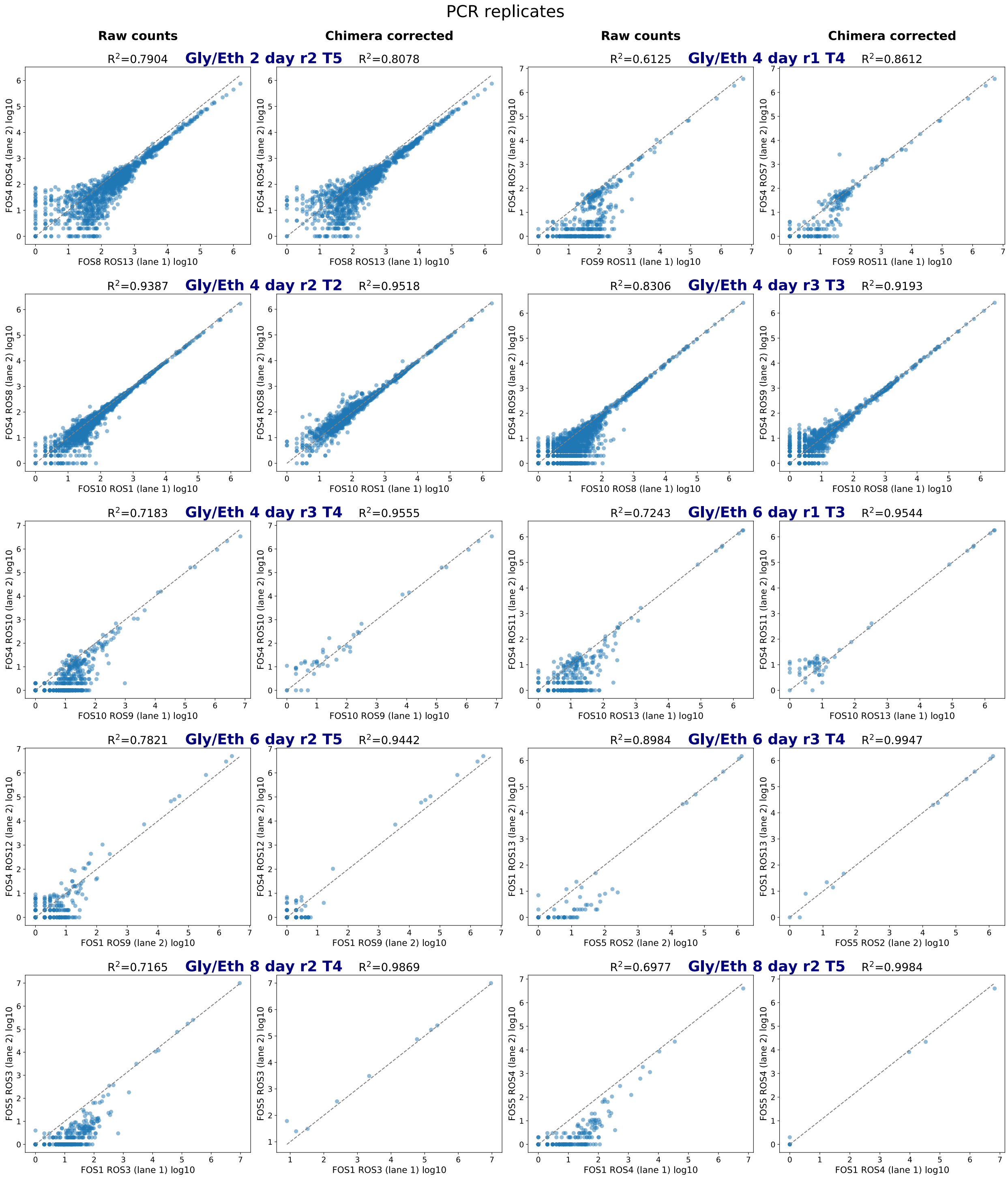

bioRxiv preprint doi: https://doi.org/10.64898/2026.03.12.711341; this version posted March 16, 2026. The copyright holder for this preprint (which was not certified by peer review) is the author/funder, who has granted bioRxiv a license to display the preprint in perpetuity. It is made available under aCC-BY 4.0 International license.

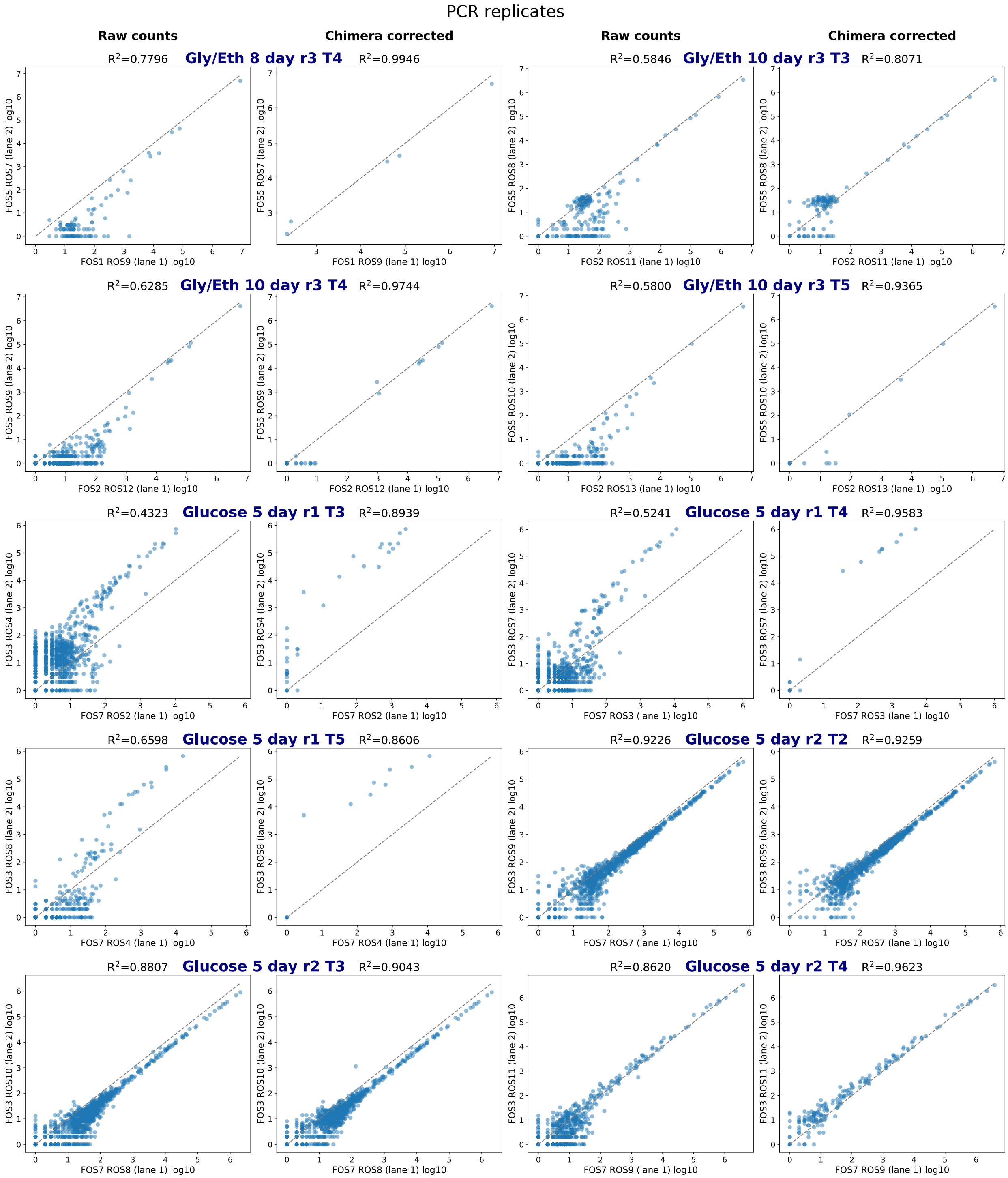

bioRxiv preprint doi: https://doi.org/10.64898/2026.03.12.711341; this version posted March 16, 2026. The copyright holder for this preprint (which was not certified by peer review) is the author/funder, who has granted bioRxiv a license to display the preprint in perpetuity. It is made available under aCC-BY 4.0 International license.

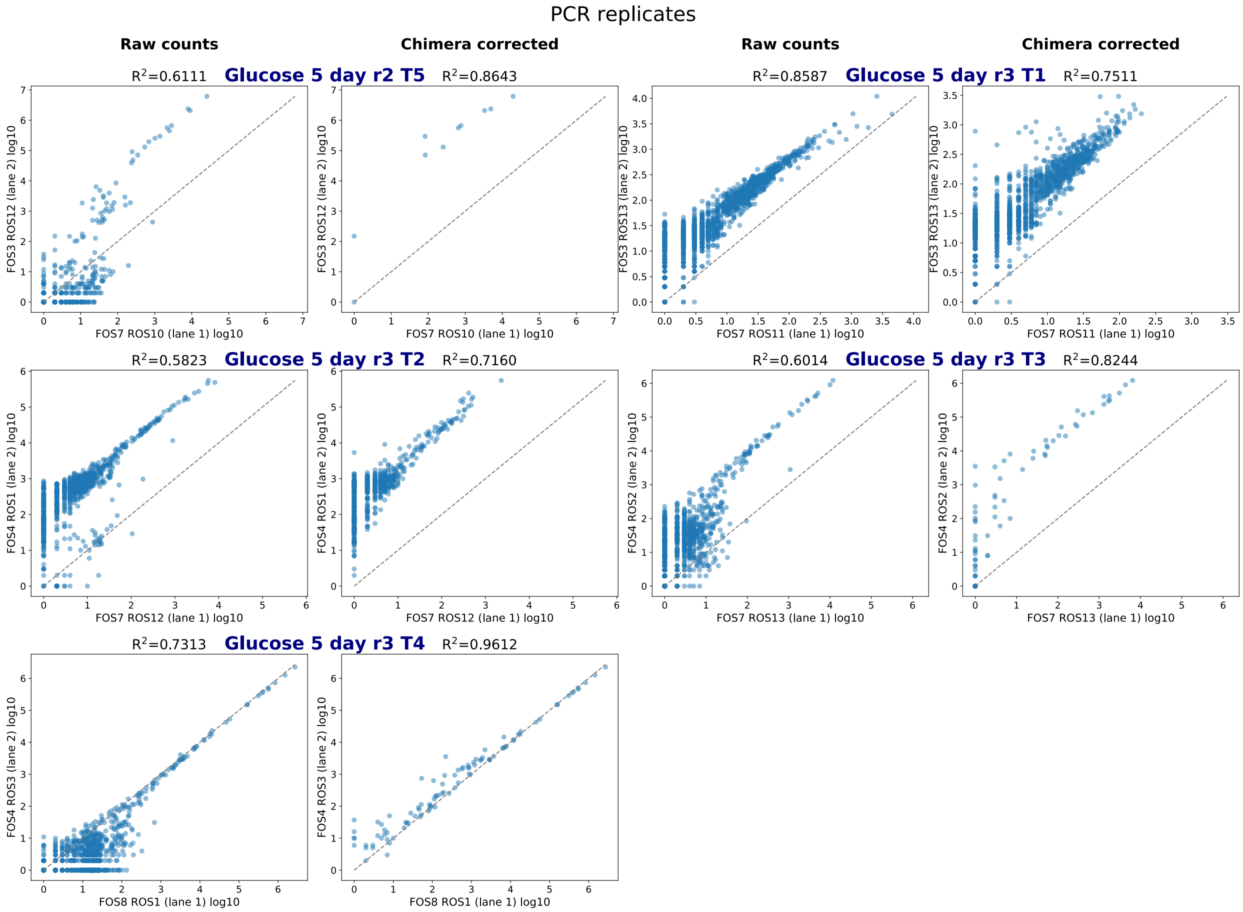

Figure S16. Technical PCR replicates are correlated. For a handful of samples we prepared a second amplicon library for quality assessment purposes. Here we show the raw and index-hopped correct counts from each of the library preparations (technical PCR replicates). Following correction for index-hopping, technical replicates are highly correlated even when one of the amplicon libraries is under-sequenced.
